# Research progress in RBC alloimmunization

**DOI:** 10.3389/fimmu.2025.1677581

**Published:** 2025-10-08

**Authors:** Zike Fu, Xinxin Hao, Wa Gao, Quishi Wang

**Affiliations:** Department of Blood Transfusion, Shengjing Hospital of China Medical University, Shenyang, Liaoning, China

**Keywords:** red blood cell alloimmunization, immune tolerance, antigen immunogenicity, transfusion immunology, sickle cell disease

## Abstract

Red blood cell (RBC) alloimmunization is a common and clinically significant immunological phenomenon in transfusion medicine, pregnancy management, and organ transplantation. It involves complex interactions between RBC antigens and the host immune system. Recent studies have revealed that RBCs are not merely passive immunological targets but also play more complex roles in the initiation and regulation of alloimmune responses. This review begins with the immunogenic properties of RBC antigens and systematically outlines the molecular mechanisms of alloimmunization, including T cell-dependent and-independent responses, functional differentiation of dendritic cells and marginal zone B cells, complement regulation, and multiple pathways of immune tolerance. On this basis, we highlight key factors influencing the occurrence of alloimmunization, such as antigen characteristics, recipient inflammatory status, donor RBC quality, underlying disease conditions, transfusion-related variables, and other potential mechanisms. Using sickle cell disease (SCD) and hemolytic disease of the newborn (HDFN) as representative models, we further explore the distinctive features and clinical implications of RBC alloimmunization in different disease contexts. This review aims to provide a systematic framework for understanding RBC-mediated immune responses and to establish a theoretical foundation for developing individualized immunomodulatory strategies.

## 1 Introduction

Alloimmunization refers to the specific immune response of an individual against alloantigens, particularly the recognition and response to RBC surface blood group antigens. Clinically, alloimmunization frequently occurs in transfusion, pregnancy, and organ transplantation, and carries significant clinical implications. In transfusion medicine, mismatches between donor and recipient RBC blood group antigens (e.g., ABO, Rh, Kell systems) can stimulate the recipient to produce alloantibodies, leading to hemolytic transfusion reactions (HTR) or delayed serologic transfusion reactions (DSTR) (1). During pregnancy, when the fetus inherits paternal RBC antigens (such as RhD) absent in the mother, the maternal immune system may generate antibodies against fetal RBCs. IgG class antibodies targeting RBC antigens can cause hemolytic disease of the newborn (HDFN), which in severe cases may result in fetal anemia, hydrops, or even death (2). In organ transplantation, RBC antigen incompatibility between donor and recipient may also trigger immune rejection, particularly in ABO-incompatible liver, kidney, and heart transplantation, where graft survival can be compromised (3–7). Interestingly, in infants and young children undergoing ABO-incompatible heart transplantation, the immaturity of the immune system, characterized by the lack of IgM^+^CD27^+^ B cells, is often associated with specific tolerance to donor ABH antigens, together with a reduced incidence of class II HLA antibodies, suggesting that ABO antigen-related tolerance may drive a systemic remodeling of alloimmune responses (8, 9). Therefore, elucidating the molecular basis and regulatory mechanisms of RBCs as targets of alloimmunization is crucial not only for improving transfusion safety and minimizing immune complications, but also for optimizing pregnancy management and transplant immunology strategies.

For a long time, RBCs were regarded solely as passive targets of the immune system, with their primary function limited to oxygen and carbon dioxide transport. However, accumulating evidence has updated this perception, suggesting that RBCs are not merely “victims” of immune responses but also active modulators of immune homeostasis. RBCs express a range of immune-related molecules on their surface, such as complement receptor 1 (CR1), CD47, and decay-accelerating factor (DAF), which participate in immune complex clearance, complement regulation, and self-recognition, thereby influencing the functions of T cells, B cells, and macrophages (10–12). In addition, RBCs can release extracellular vesicles (RBC-derived extracellular vesicles, REVs), which contribute to inflammatory regulation and immune signaling (13). These findings indicate that RBCs participate in alloimmune responses not only as antigenic targets but also as immunoregulatory players via surface receptors, vesicle secretion, and pattern recognition properties, thereby exerting functions far beyond traditional understanding.

This review aims to provide a systematic overview of the central roles and molecular mechanisms of RBCs in alloimmunization, with a focus on their function as antigenic targets mediating immune recognition and pathological injury. By doing so, we seek to enhance the understanding of the multifaceted roles of RBCs in alloimmunity and to lay the groundwork for developing targeted immunomodulatory strategies against RBC-specific antigens.

## 2 Molecular basis of red blood cell immunogenicity

RBC surface antigens are the central triggers of alloimmune responses. To date, the International Society of Blood Transfusion (ISBT) has recognized 48 blood group systems comprising more than 300 distinct antigenic molecules. Among these, the ABO, Rh, Kell, Duffy, Kidd, and MNSs systems are of greatest clinical importance (14).

The immunogenicity of RBC surface antigens depends on their chemical properties, molecular structures, epitope accessibility, and the immunological status of the host. Significant structural differences exist among blood group antigens, directly influencing both their immunogenic strength and clinical relevance. Protein antigens, such as RhD and Kell, generally exhibit higher immunogenicity than carbohydrate antigens, such as ABO and Lewis. Protein antigens can more effectively stimulate T cell-dependent immune responses, induce memory B cell formation, and elicit stronger antibody production upon re-exposure. Within the Rh system, antigens such as RhD are transmembrane proteins, whose immunogenicity is determined not only by protein conformation but also by epitope density. RhD-mediated hemolytic disease of the fetus and newborn (HDFN) is the most common and severe form of alloimmune hemolytic disease, with greater clinical severity compared with cases induced by other blood group systems, such as Kell, Duffy, or Kidd (15). Notably, in the absence of prophylaxis, approximately 56% of RhD-sensitized pregnant women experience progressively aggravated HDFN in subsequent pregnancies, a rate significantly higher than that observed with alloantibodies from other blood group systems (16, 17).

In contrast, ABO blood group antigens are carbohydrate-based structures that usually induce immune responses through T cell-independent pathways, predominantly generating natural IgM antibodies and lacking classical immunological memory (18–20). However, ABO antibodies are not limited to IgM; they also include IgG and IgA. Some of these antibodies may be generated through T cell-dependent mechanisms following ABO-incompatible antigen exposure, such as mismatched transfusion or pregnancy. Clinically, ABO antibodies are highly significant, as both IgM and IgG subclasses can effectively activate complement and cause acute hemolytic reactions (21, 22). ABO antigens consist of glycan chains modified by glycosyltransferases, and their immunogenicity is determined by subtle differences in terminal sugars. For example, the A antigen carries N-acetylgalactosamine, whereas the B antigen carries galactose. These structural differences explain why individuals lacking the corresponding antigen develop natural antibodies (23, 24). Because ABO antigens are abundantly expressed on the RBC surface, even a small number of incompatible RBCs entering the circulation can trigger robust complement activation and acute hemolysis (25).

Beyond the ABO and Rh systems, other blood group antigens such as Kell, Duffy, and Kidd also display important immunological properties. The Kell antigen (K1/K2), a zinc endopeptidase, exhibits very high immunogenicity second only to RhD. Exposure through transfusion or pregnancy readily induces IgG alloantibody production, which is strongly associated with severe hemolytic reactions (26, 27). The Duffy antigen (Fy^a^/Fy^b^) functions as a chemokine receptor; its polymorphisms not only predispose multiply transfused patients to alloimmunization but also influence susceptibility to Plasmodium infection (28). The Kidd antigen (Jk^a^/Jk^b^), which is expressed on both RBCs and renal tissue, is clinically notable because its antibodies can cause delayed hemolytic transfusion reactions (DHTR), a process linked to memory B cell activation and secondary immune response amplification (29).

## 3 Immunological mechanisms of red blood cell alloimmunization

The initiation of red blood cell (RBC) alloimmunization is a complex immunological process involving multiple innate and adaptive immune cells and molecular pathways. This process begins with the recognition of damage-associated molecular patterns (DAMPs) by the innate immune system. DAMPs are endogenous molecules released during cellular stress, injury, or death, including mitochondrial DNA, heme, adenosine triphosphate (ATP), heat shock proteins, and high-mobility group box 1 (HMGB1). While these molecules perform physiological functions intracellularly, once released into the extracellular milieu they are recognized by pattern recognition receptors (PRRs), thereby converting into danger signals that activate inflammatory responses (30, 31). During storage, RBCs progressively release DAMPs such as ATP and heme, which activate the nucleotide-binding oligomerization domain-like receptor family pyrin domain-containing 3 (NLRP3) inflammasome and stimulate macrophages and dendritic cells (DCs) to secrete pro-inflammatory cytokines, thereby amplifying immune responses (32, 33).

When allogeneic RBCs enter the circulation, their surface antigens are recognized by host antigen-presenting cells (APCs), including DCs and macrophages, through PRRs. These APCs can be activated by DAMPs through several PRR-dependent pathways. For example, Toll-like receptors (TLRs) trigger myeloid differentiation primary response 88 (MyD88)-orTIR-domain-containing adapter-inducing interferon-β (TRIF)-dependent signaling to induce inflammatory cytokines and type I interferons (IFN-I), whereas the NLRP3 inflammasome senses extracellular ATP, uric acid, or heme to promote caspase-1 activation and subsequent release of interleukin (IL)-1β and IL-18. In addition, intact DAMP-containing units derived from stored RBCs can be engulfed by CD11b^+^Ly6C^+^F4/80^+^ macrophages, leading to the secretion of pro-inflammatory mediators that further promote conventional dendritic cell (cDC) activation and maturation (32, 34–37). This recognition event ultimately activates APCs and drives the secretion of pro-inflammatory cytokines, such as IL-6 and tumor necrosis factor-alpha (TNF-α), along with chemokines that establish an inflammatory microenvironment.

Adaptive immunity subsequently dominates the alloimmune response. Activated APCs migrate to secondary lymphoid organs, where processed alloantigens are presented via major histocompatibility complex (MHC) class II molecules to CD4^+^ T cells. Under the influence of costimulatory signals and cytokines, naïve T cells differentiate into T helper (Th) subsets, particularly Th2 cells. These Th cells secrete IL-4 and IL-5, which promote B cell activation, proliferation, and differentiation into plasma cells, leading to the production of alloantigen-specific antibodies, primarily of the immunoglobulin G (IgG) class (38). Protein antigens, in particular, are capable of eliciting robust T cell-dependent responses and long-lived immunological memory. Consequently, this process is especially prominent in immune responses to protein antigens such as RhD (18).

Recent studies have shown that stored RBCs can activate cDCs via MyD88-dependent but TRIF-independent pathways (39), suggesting that RBCs are not merely passive antigen carriers but can function as immune adjuvants. This activation promotes the maturation of cDC2 subsets, which in turn efficiently initiate CD4^+^ T cell responses and drive the generation of RBC-specific alloantibodies. Within this process, 33D1^+^ cDC2s have a unique capacity to present RBC-derived antigens to splenic CD4^+^ T cells. In contrast, other cDC subsets such as XCR1^+^ cDC1s, although capable of comparable antigen uptake and presentation *in vitro*, are ineffective at inducing antibody responses *in vivo*. This highlights 33D1^+^ cDC2s as the indispensable DC subset for driving alloantibody responses against HOD RBCs (40). The spleen plays a central role in this process, functioning not only as the primary site of RBC antigen presentation and T cell priming but also as a specialized microenvironment for immune cell interactions (41).

The MyD88 signaling pathway is critical for regulating T cell responses. Although MyD88 deficiency does not directly affect T cell survival or regulatory T cell (Treg) development, it markedly reduces the ability of DCs to produce pro-inflammatory cytokines, thereby impairing T cell activation and expansion. This results in fewer effector T cells, weaker antigen-specific responses, and increased susceptibility to Treg-mediated suppression (42). Moreover, intrinsic MyD88 signaling in T cells is essential for follicular helper T cell (Tfh) differentiation, suppressing Tfh polarization while promoting effector T cell fate (43). Additional studies have demonstrated that interleukin-6 receptor alpha (IL-6Rα) signaling drives Tfh differentiation and antibody production in murine models of RBC alloimmunization, underscoring its upstream regulatory role in CD4^+^ T cell-mediated immune responses (44).

Beyond T cell-mediated adaptive immunity, RBC alloimmunization can also proceed through T cell-independent mechanisms. Studies using the Kell (KEL) antigen model have shown that transfusion with KEL-expressing RBCs induces robust humoral immune responses in the absence of MHC class II-restricted CD4^+^ T cell help. Marginal zone (MZ) B cells are central to this process: depletion of MZ B cells completely abolishes anti-KEL antibody production, whereas removal of follicular B cells has little effect. This suggests that MZ B cells can directly recognize RBC antigens and generate IgG antibodies independently of T cell help (45, 46). These findings challenge the classical paradigm that humoral immunity is strictly dependent on T cell assistance and highlight alternative pathways of B cell activation by RBC antigens.

From an antigenic perspective, certain carbohydrate or highly repetitive RBC antigens, such as ABO antigens, are classified as type II T cell-independent antigens (TI-2). Their repetitive epitope structures allow for “zipper-like” B cell receptor (BCR) crosslinking, which directly activates B cells in the absence of T cell help. Unlike type I T cell-independent antigens (TI-1), such as lipopolysaccharide (LPS), which rely on TLR signaling to induce polyclonal activation, TI-2 antigens preferentially induce low-affinity IgM. Under certain conditions, however, class switching can occur, leading to IgG production (47–49). MZ B cells and B-1 cells are the primary responders to TI-2 antigens, rapidly differentiating into IgM-secreting plasma cells or producing limited, short-lived memory responses (50–54).

It is important to note that although MZ B cells can be activated in the absence of direct CD4+ T cell help, the process of switching from IgM to IgG production still requires support from other immune cells. First, NKT cells can directly interact with MZ B cells through CD1d and, under inflammatory conditions, rapidly secrete cytokines such as IL-4 and IFN-γ, which provide critical signals for MZ B cell activation and class-switch recombination (CSR) (49). Furthermore, a specialized subset of splenic neutrophils, termed B cell–helper neutrophils (NBH), release BAFF, APRIL, and IL-21, thereby enhancing MZ B cell survival, proliferation, and antibody secretion. On this basis, dendritic cells and macrophages, upon pathogen-associated stimulation, can also produce similar factors, further expanding and consolidating this supportive network (20). In other words, the generation of IgG by MZ B cells does not rely solely on their intrinsic capacity, but instead depends on their collaboration with multiple innate immune cell types and the cytokine milieu they establish, which collectively enable efficient class switching.

Accordingly, antibodies induced by RBC antigens may arise as “natural antibodies,” generated primarily by B-1 cells during early life in response to cross-reactive antigens from the gut microbiota (with anti-A and anti-B blood group antibodies as classical examples), or as “acquired antibodies,” produced by MZ B cells following later exposure to cross-reactive antigens or alloantigens on transfused RBCs. This dual origin explains why individuals often carry low titers of RBC antibodies even in the absence of transfusion or pregnancy. Although MZ B cells can initiate IgG production without direct T cell help, they still rely on the supportive cytokine milieu provided by NKT cells, neutrophils, and dendritic cells.

RBC-induced immune responses also differ significantly from those elicited by conventional protein antigens. For example, the generation of anti-KEL antibodies not only requires MZ B cells but also depends on type I interferon signaling mediated through the interferon-alpha/beta receptor (IFNAR). Effective B cell responses to RBC antigens require IFNAR-mediated signal transduction, highlighting the regulatory role of innate signals in RBC immunogenicity. IFNAR signaling influences not only B cell activity but also modulates cDC activation, thereby shaping T cell-dependent immunity. In models of K1 antigen immunization under inflammatory conditions, CD8α^+^ cDCs have been shown to produce IFN-α/β, which is sufficient to induce alloimmunization even in the absence of polyinosinic:polycytidylic acid [poly(I:C)] (55–58).

The complement system also contributes dual regulatory roles in this process. On the one hand, complement component C3 modulates both the magnitude and pathway of the immune response, potentially acting as a “molecular switch” between T cell-dependent and T cell-independent routes. On the other hand, C3 deposition can reduce antigen availability on the RBC surface, thereby limiting antibody formation through an “antigen shielding” effect. This mechanism preserves RBC structural integrity while providing a protective strategy against excessive immune activation. Importantly, the immunomodulatory effects of C3 depend on its activation threshold, with significant influence only observed under conditions of robust complement activation. Furthermore, expression of complement receptors CR1 and CR2 on hematopoietic cells is essential for C3-mediated regulation of antibody responses (59).

**Figure 1:**
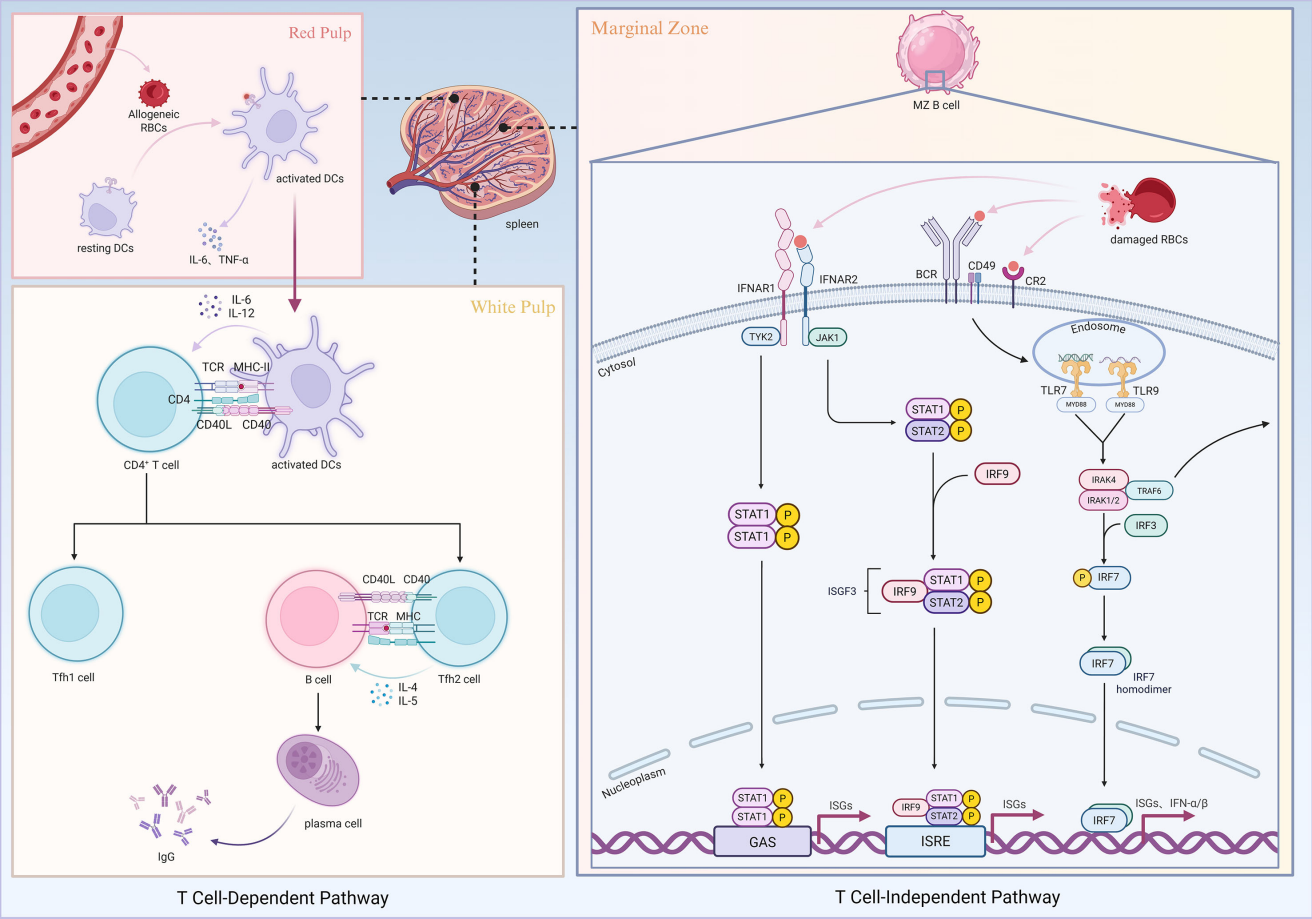
Mechanisms of RBC alloimmunization. Created in https://BioRender.com.

Taken together, RBC alloimmunization does not result from a single mechanism but rather from the coordinated interplay of multiple pathways, including DAMP-mediated innate receptor signaling, DC activation, T cell regulation, MZ B cell responses, IFNAR signaling, and complement-mediated antigen modulation (**Figure 1**). Unlike conventional protein antigens, RBCs engage unique modes of immune activation that underscore the inherent complexity of transfusion-related immunity, providing essential insights for developing safer transfusion practices and novel immunomodulatory strategies.

## 4 Regulatory mechanisms of immune tolerance to red blood cell antigens

Although RBCs express a variety of alloantigens on their surface, not all transfusion recipients develop immune responses, suggesting the existence of a certain degree of immune tolerance to RBC antigens. This phenomenon is particularly evident in patients who undergo multiple transfusions without developing detectable antibodies. The relatively low immunogenicity of RBCs partially arises from their biological features: they lack major histocompatibility complex (MHC) molecules and costimulatory molecules, do not express Toll-like receptors (TLRs), and do not secrete immune-activating cytokines. As such, RBCs alone are often insufficient to function as “complete antigens” capable of eliciting adaptive immune responses. However, this low immunogenicity is not absolute. In the presence of inflammation or danger signals, RBC antigens can still induce robust immune responses. In the absence of such signals, RBC antigens may instead be perceived as “self-like” antigens, leading to immune ignorance or the induction of tolerance.

On the one hand, the inflammatory context of innate immunity is a key determinant of whether RBC antigens elicit immunity or tolerance (60). Under non-inflammatory conditions, APCs such as DCs often remain in a quiescent or immature state. When these cells capture RBC antigens, they are more likely to induce T cell anergy rather than activation and proliferation. Studies have shown that in the absence of MyD88 signaling, DCs fail to upregulate costimulatory molecules such as CD80 and CD86 and thus cannot effectively activate CD4^+^ T cells even when exposed to RBC antigens, thereby promoting the establishment of peripheral tolerance (42).

On the other hand, regulatory T cells (Tregs) play a critical role in RBC-associated tolerance. By secreting immunosuppressive cytokines such as IL-10 and transforming growth factor-beta (TGF-β), Tregs inhibit effector T cell activation and expansion, and directly modulate DC maturation and antigen presentation. Evidence suggests that in a tolerogenic environment, Tregs exert stronger suppression on effector T cells (61, 62), and this suppressive effect can be further enhanced by factors such as MyD88 deficiency or increased complement component C3 activity (42, 59).

The complement system may also contribute to tolerance induction. C3 deposition can mask or internalize RBC surface antigens, thereby reducing their immunological accessibility and limiting their efficiency of presentation in secondary lymphoid organs (59). Moderate complement activation may favor “silent clearance” rather than immunogenic stimulation. In addition, the expression of complement receptors CR1 and CR2 on hematopoietic cells is closely associated with antigen presentation. Their absence or dysfunction may prevent antigens from effectively entering adaptive immune pathways, resulting in immune ignorance (46).

In summary, tolerance to RBC antigens arises from multiple interconnected mechanisms, encompassing both the intrinsic “stealth” and low-inflammatory nature of the antigens themselves and the regulatory functions of Tregs, regulatory B cells (Bregs), complement, and DCs. A deeper understanding of these pathways not only explains the relatively low rate of alloantibody formation observed in clinical practice but also provides a theoretical foundation for developing tolerance-inducing strategies, such as RBC antigen-specific tolerogenic vaccines or pretransfusion tolerance-induction protocols.

## 5 Factors influencing red blood cell alloimmunization

**Figure 2:**
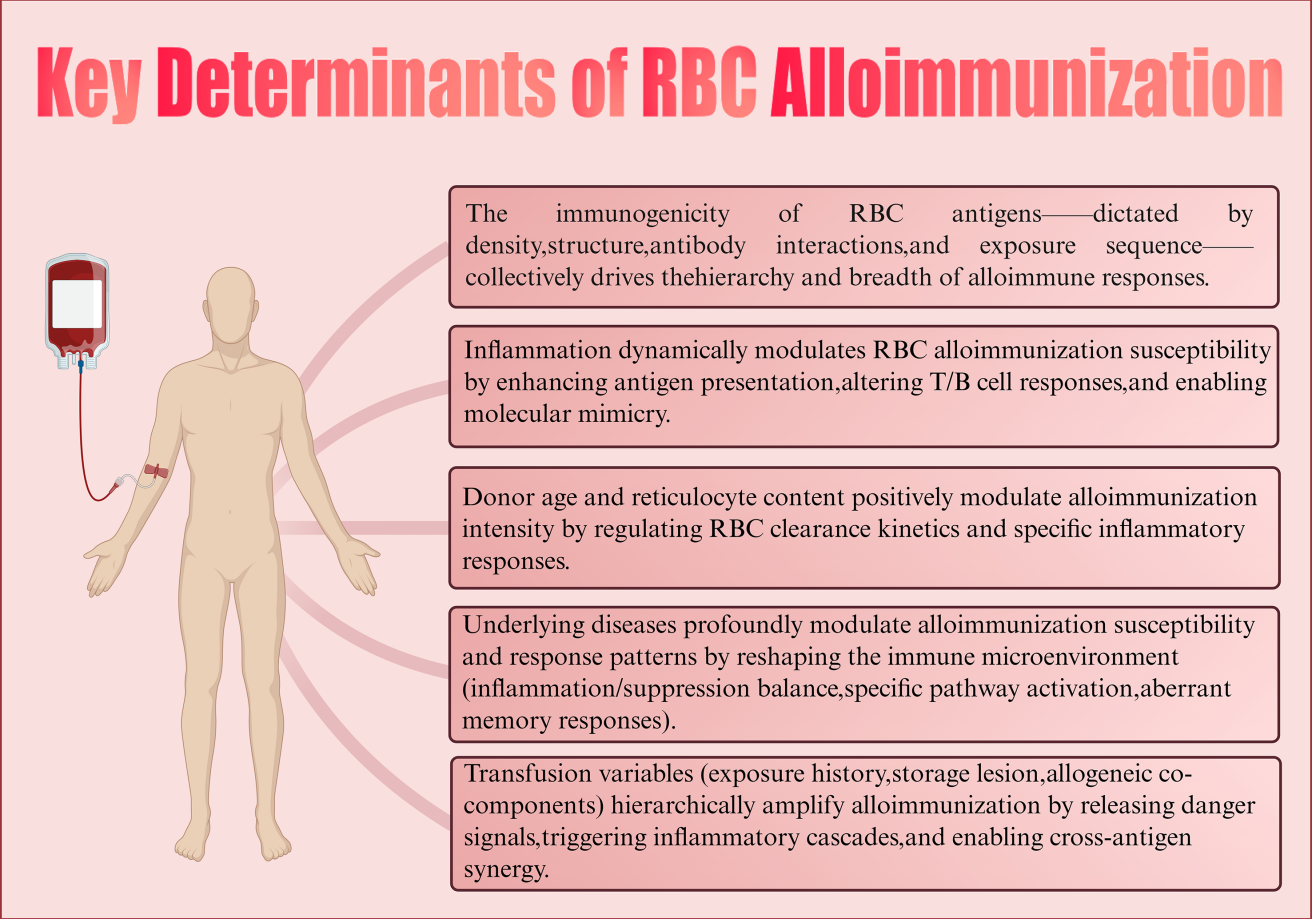
Key determinants of RBC alloimmunization. Created in https://BioRender.com.

The determinants of alloimmunization are multifactorial. Current research suggests that at least three major aspects are involved: antigenic disparities between donor and recipient RBCs, the immunological status of the recipient, and the immunomodulatory effects of transfused allogeneic RBCs on the recipient’s immune system (63, 64). Beyond these, additional variables such as genetic background, underlying disease, inflammatory environment, transfusion history, and storage conditions of transfused RBCs may also influence the likelihood and intensity of alloimmune responses (65) (**Figure 2**). As these factors are often interwoven, the mechanisms of alloimmunization remain complex and incompletely understood. Elucidating these determinants is critical for understanding the pathogenesis of RBC sensitization, optimizing transfusion strategies, and preventing alloimmune reactions.

### 5.1 Antigen characteristics and the basis of immunogenicity

The intrinsic properties of RBC antigens play a central role in alloimmunization. Their immunogenicity is determined by multiple factors, including antigen density, epitope structure, antigen type, and the sequence of antigen exposure.

Antigen density is one of the most important determinants of immune responses. Studies have demonstrated that exposure to RBCs with low antigen density can induce tolerance, significantly reducing subsequent immune responses to high-density RBCs. This suggests that antigen density not only influences the magnitude of the primary response but may also shape long-term immunological outcomes (66). Antigens with low copy numbers are more likely to promote tolerance, whereas high-copy antigens require more potent regulatory mechanisms, such as higher doses of blocking antibodies, to suppress immune activation (67). Importantly, antigen density varies markedly across blood group systems, ranging from only a few copies to more than one million per RBC, which may partly explain the substantial differences in immunogenicity among distinct antigens.

Antigen structure also contributes significantly to immunogenicity. Different RBC antigens vary in their ability to elicit humoral immune responses (46, 68, 69). Interestingly, highly immunogenic antigens such as K, Jk^a^, Lu^a^, and E often display fewer B cell epitopes, whereas less immunogenic antigens such as Fy^a^, S, C, and c possess a greater number of epitopes (70). This “less is more” phenomenon suggests that structurally streamlined antigens may more effectively trigger immune recognition.

Antibody-antigen interactions can further modulate antigen immunogenicity. Certain alloantibodies are capable of converting otherwise tolerogenic stimuli into immunogenic ones. This enhancement is antigen-specific and influenced by antibody isotype; for instance, IgG2c has been shown to augment the immunogenicity of RBC antigens (71).

The order of antigen exposure may also shape subsequent immune responses to heterologous antigens. Evidence indicates that CD4^+^ T cells primed by a specific RBC antigen can enhance humoral responses to other antigens, suggesting cross-regulatory interactions (72). This “priming effect” may help explain why some patients develop progressively broader sensitization following multiple transfusions and underscores the decisive role of the initial antigen encounter. Of note, RBC alloimmunization is often associated with human leukocyte antigen (HLA) alloimmunization, suggesting that the overall activation state and regulatory capacity of the immune system may broadly influence responses to RBC antigens (73, 74).

In summary, the density, structural features, biological properties, and exposure dynamics of RBC antigens collectively determine their immunogenicity. These factors are key drivers of the initiation and evolution of alloimmunization.

### 5.2 Impact of recipient immune and inflammatory status

The immune status of the recipient, particularly the presence of inflammation, is a major determinant of whether RBC alloimmunization occurs. Both clinical and experimental studies have consistently demonstrated that inflammation markedly enhances the immunogenicity of alloantigens, converting otherwise tolerant recipients into responders (60).

In pediatric patients with sickle cell disease (SCD), it has been shown that even when transfused with Rh- and Kell-negative or fully matched RBCs, the risk of alloimmunization increases significantly if inflammation is present at the time of transfusion (75). Similarly, murine studies have revealed that transfusion during poly(I:C)-induced viral-like inflammatory states promotes enhanced alloantibody production (76). Inflammatory conditions augment the phagocytosis of RBCs by splenic dendritic cells (DCs), accompanied by upregulation of MHC class II and costimulatory molecules such as CD86. This process is critical for antigen presentation and T cell activation. Specifically, CD8^+^ and CD11b^+^ DC subsets play essential roles in stimulating alloantigen-specific CD4^+^ T cell proliferation and immune synapse formation, thereby triggering antigen-specific immune responses against RBCs (77).

Clinical data further indicate that patients with prolonged fever or bacterial infections exhibit higher rates of alloimmunization, and viral infections appear to exert similar effects (58). For example, influenza infection not only enhances germinal center B cell responses but also amplifies T follicular helper (Tfh) cell responses to transfused RBC antigens, thereby increasing antigen-specific cytokine release and promoting alloantibody production. The interferon-alpha/beta (IFN-α/β) signaling pathway plays a pivotal role in this process (57). Additionally, certain pathogens share peptide sequence homology with RBC antigens, potentially activating pre-existing CD4^+^ T cells through molecular mimicry, which may then mediate alloantibody formation upon subsequent RBC transfusion. Such mechanisms are difficult to detect with routine serological screening in blood banks, but they may help explain interindividual variability in alloimmune responses (78).

Notably, the impact of infection on alloimmunization is not uniform. For instance, the risk of alloimmunization appears to decrease during Gram-negative bacteremia, highlighting the complexity of immunoregulatory mechanisms and interindividual variability (58).

In summary, the inflammatory status of the recipient profoundly shapes RBC alloimmunization by modulating APC function, T cell activation, and the cytokine milieu. The type of infection, the intensity of inflammation, and its temporal relationship to transfusion collectively determine the immune system’s responsiveness to RBC antigens, making it a critical factor in alloimmunization.

### 5.3 Donor red blood cell characteristics and their modulatory effects on immune responses

Donor-related characteristics may also influence the occurrence of RBC alloimmunization. Evidence suggests that donor sex, age, and racial/ethnic background are associated with the fragility and hemolysis propensity of stored RBCs. For instance, RBCs from male donors generally display higher rates of hemolysis under storage and osmotic stress conditions compared to female donors. Similarly, RBCs from Asian and African American donors exhibit greater spontaneous or stress-induced hemolysis compared to those from White donors. The effects of donor age appear more complex: while RBCs from younger and middle-aged donors tend to have higher storage hemolysis rates, some studies report reduced hemolysis in RBCs from older donors (79, 80). These findings suggest that donor characteristics may modulate RBC stability during storage and clearance in recipients, thereby influencing immunogenicity.

Another important factor is the proportion of reticulocytes in the transfused RBC product. Studies in murine models have shown that transfusion of RBCs enriched in reticulocytes enhances alloimmune responses and alloantibody production in a dose-dependent manner. This is accompanied by accelerated clearance of RBCs from circulation and elevated levels of proinflammatory cytokines such as MCP-1, CXCL1, CXCL10, and interferon-gamma (IFN-γ), reflecting a specific and selective inflammatory response (81). Notably, even low proportions of reticulocytes are sufficient to augment immune responses, underscoring their high sensitivity. Further investigations demonstrated that reticulocyte-enriched RBCs enhance phagocytic activity across major B cell subsets, thereby generating a stronger immunostimulatory environment. This augmented immune response is independent of TLR4 signaling and not attributable to bacterial endotoxin contamination, confirming the intrinsic role of reticulocytes in promoting alloimmunization (81).

In summary, donor-related factors such as sex, age, ethnicity, and reticulocyte content may collectively shape the immunogenicity of RBCs. However, direct causal relationships between these variables and alloimmunization risk remain insufficiently established in clinical studies, and inconsistent results have been reported across different cohorts. Further research is needed to clarify the true contribution of donor characteristics to alloimmunization.

### 5.4 Impact of underlying diseases on red blood cell alloimmunization

Underlying diseases may influence the induction of RBC alloimmunization by modulating B cell activation, antigen processing, or the immune microenvironment. In transfusion-dependent thalassemia patients, the prevalence of RBC alloimmunization can reach 11.4% (82). In patients with sickle cell disease (SCD), the rate of alloantibody formation is even higher; however, some studies suggest the presence of intrinsic immunoprotective mechanisms. Heme, a prototypical damage-associated molecular pattern (DAMP), is released from RBCs during chronic hemolysis. It can directly promote inflammation by oxidizing lipids, proteins, and DNA, while also regulating immune responses via signaling pathways (83, 84).

In non-alloimmunized SCD patients, heme has been shown to suppress B cell differentiation into plasmablasts and plasma cells through inhibition of the DOCK8/STAT3 pathway, thereby dampening humoral immunity (83). Concurrently, heme modulates DC maturation via the TLR4-MyD88-NF-κB axis, downregulating CD83 and proinflammatory cytokine expression and limiting Th1 differentiation. This protective mechanism is absent in a subset of alloimmunized SCD patients, in whom defective inhibitory signaling leads to enhanced DC activation and subsequent alloantibody production (84, 85). In contrast, in transfusion-dependent thalassemia, alloimmunization has been linked to altered DOCK8/STAT3 signaling, suggesting that the hemolytic microenvironment plays a central role in shaping alloimmunization risk. By comparison, in other contexts of chronic inflammation or immune dysregulation, the risk of RBC alloimmunization is significantly increased.

Heme metabolites also exert profound immunoregulatory effects. Heme oxygenase-1 (HO-1) catabolizes heme to produce carbon monoxide (CO) and biliverdin, both of which have anti-inflammatory and antioxidant properties. These metabolites polarize macrophages toward M2 or M-hem anti-inflammatory phenotypes, reduce proinflammatory cytokine production, and enhance phagocytic capacity (86, 87). CO additionally inhibits inducible nitric oxide synthase (iNOS) activity and NO production, while upregulating indoleamine 2,3-dioxygenase (IDO), thereby reinforcing immunosuppressive pathways. Treg cells act synergistically with HO-1 and IDO, becoming upregulated in inflammatory or interferon-gamma (IFN-γ)-rich conditions, where they suppress effector cell proliferation and induce apoptosis, collectively promoting immune tolerance (88, 89).

Nevertheless, the effects of heme are dualistic. Excess accumulation can drive inflammation through reactive oxygen species (ROS) production, activation of the NLRP3 inflammasome, and induction of neutrophil extracellular traps (NETs), leading to endothelial activation and tissue injury (84). In SCD, heme has been shown to induce ROS generation and NF-κB activation via protein kinase C (PKC) and spleen tyrosine kinase (Syk) signaling, thereby exacerbating vasculopathy and inflammation (90). Although HO-1 activity provides partial counter-regulation, persistent hemolysis and immune dysregulation can overwhelm this protective effect (86, 87, 91–94).

SCD and hemolytic disease of the fetus and newborn (HDFN) are representative models of RBC alloimmunization. SCD patients are considered a high-risk population due to their chronic inflammatory state and repeated transfusions (95, 96), while HDFN exemplifies alloimmunization through fetomaternal antigen exposure (97). The prevalence of alloimmunization in SCD is markedly higher than in other chronically transfused conditions such as thalassemia or chronic kidney disease (98, 99). This heightened risk is not fully attributable to transfusion frequency but is strongly shaped by the immune background. SCD patients frequently exhibit chronic low-grade inflammation, impaired Treg function, enhanced Tfh activity, increased B cell propensity toward high-affinity plasma cell differentiation and altered antigen presentation by monocytes and DCs—all contributing to a host environment primed for immune induction (100–105). While Treg, IDO, and HO-1 expression are regarded as potential protective mechanisms (86, 87, 91–94), dysregulation of heme/TLR4 signaling and HO-1 pathways in SCD patients often renders these mechanisms insufficient, partially explaining their high alloimmunization risk (85).

SCD is also characterized by delayed hemolytic transfusion reactions (DHTRs) and their severe form, hyperhemolysis syndrome (HHS) (106–109). These events present with post-transfusion hemoglobin decline, reticulocytopenia, and often absent or delayed antibody detection, leading to frequent misdiagnosis as vaso-occlusive crises and delayed management (108, 110). Emerging evidence suggests that such reactions may involve mechanisms beyond alloantibodies, including dysregulated cytokine networks and T cell-myeloid cell-driven amplification loops (111, 112). Some patients also display prolonged periods of “antibody silence” following initial sensitization, but upon re-exposure, they develop rapid and robust immune responses—so-called “memory responses.” This may reflect heightened T cell help and imbalanced reactivation of memory responses (102, 113, 114).

By contrast, HDFN arises primarily in the context of maternal-fetal immune interactions. Clinical data indicate that the Rh system (particularly D, C, and E antigens) and Kidd system are the most frequent causes of HDFN. Among these, anti-D-mediated HDFN remains the most severe and globally prevalent form (15–17, 27, 115, 116), with incidence varying across populations (117). Although anti-D immunoglobulin (RhIg) prophylaxis has dramatically reduced its incidence, severe cases still occur in multiple pregnancies, suboptimal prophylaxis, or high-risk individuals (118). Anti-K-associated HDFN can present earlier and with more severe anemia and hydrops fetalis, often requiring intrauterine transfusion. Postnatally, affected neonates may exhibit persistent anemia and hyperbilirubinemia, underscoring the strong suppressive effect of anti-K antibodies on fetal erythropoiesis (27).

Autoimmune diseases, particularly systemic lupus erythematosus (SLE), are another major background factor predisposing to the production of multiple or rare RBC alloantibodies (119). Type I interferons (IFN-α/β) play a central role in SLE pathogenesis and autoantibody generation, and they also regulate RBC alloimmunization (120). Through IFNAR1 signaling, IFN-α/β promote B cell differentiation into germinal center and antibody-secreting cells, serving as key drivers of anti-KEL IgM production. Lupus mouse models further confirm that IFN-α/β-dependent inflammation amplifies immune responses to RBC antigens, highlighting their importance in autoimmunity-associated alloimmunization (121).

Collectively, these findings suggest that disease states are not merely background conditions for RBC alloimmunization but actively shape susceptibility and response heterogeneity through disease-specific molecular mechanisms.

### 5.5 Transfusion-related variables influencing immune responses

Transfusion is the direct trigger of RBC alloimmunization, with multiple associated factors influencing the magnitude of immune risk. Patient age at first transfusion is a critical determinant, with those older than two years at initial transfusion showing a significantly higher likelihood of alloantibody formation (122). The history of prior RBC transfusions and cumulative transfusion volume are also major predictors of alloimmunization (123, 124), underscoring the sensitizing effect of repeated antigen exposure.

Beyond transfusion frequency, the storage condition of RBC units is another important variable affecting immunogenicity. In animal models, transfusion of leukoreduced HOD RBCs stored for 14 days (to simulate human storage conditions) induces stronger alloantibody responses compared to fresh RBCs, along with rapid cytokine elevations (IL-6, KC, and MCP-1) within 90 minutes of transfusion (125–127). By contrast, fresh RBCs induce minimal inflammation and may even attenuate responses when co-transfused with stored RBCs, suggesting that storage-induced alterations enhance immunogenicity, whereas fresh RBCs may have immunomodulatory potential. Importantly, mitochondrial DNA (mtDNA) DAMPs have been detected in stored RBC suspensions, cryoprecipitate, and platelet products. These may act as secondary inflammatory stimuli that not only promote alloimmunization but also contribute to delayed phenotypes of transfusion-related acute lung injury (TRALI) (128).

Further studies indicate that RBCs pretreated with phenylhydrazine or heated to 50 °C undergo accelerated clearance *in vivo*, leading to stronger inflammatory responses and higher alloantibody levels. This “rapid clearance” increases antigen load and triggers DAMP release, activating innate immune pathways and amplifying humoral immunity (127). *In vivo* and *in vitro* models confirm that this mechanism is associated with high MCP-1 release, cytokine storms, and hemolytic transfusion reactions (111, 112, 129).

Clinically, however, most studies have not found a clear correlation between storage duration and alloimmunization risk (130). This aligns with the ability of healthy individuals to tolerate moderate levels of RBC breakdown products such as bilirubin and iron. Nevertheless, “older” RBCs approaching the 42-day storage limit may accumulate structural and functional abnormalities, increasing inflammatory and sensitizing potential—an effect supported by experimental but not yet clinical evidence (131, 132). Thus, while laboratory data suggest that storage lesions enhance RBC immunogenicity, clinical investigations have yet to confirm this association, likely due to patient heterogeneity, underlying disease, and complex transfusion variables (133). Clarifying the clinical impact of storage duration requires systematic studies to determine whether experimental findings can be translated into practice. Nonetheless, current data provides theoretical support for optimizing transfusion strategies and limiting storage time.

It is also noteworthy that different blood components may exert synergistic effects on alloimmunization. In murine studies, platelet transfusion from wild-type donors enhanced humoral responses to RBC antigens such as KEL, even in recipients who were otherwise non-responders to RBC transfusion alone (134). This effect was independent of platelet-derived CD40L, suggesting alternative pathways through which platelets contribute to the initiation and regulation of RBC alloimmunization.

## 6 Conclusion

RBC alloimmunization is a prototypical adaptive immune process driven by dynamic and complex interactions among antigens, recipients, and environmental factors. Advances in the structural and immunological characterization of RBC antigens have broadened our understanding of their roles in immune regulation. Beyond the classical T cell-dependent pathways, non-T cell-dependent mechanisms, DC subset specialization, and complement-mediated regulation have also been revealed. Meanwhile, non-antigenic factors such as chronic inflammation, storage lesions, and host HLA background are increasingly recognized as critical contributors to alloimmunization.

Clinically, RBC alloimmunization complicates transfusion management, exacerbates pregnancy-related complications, and increases the risk of graft rejection in transplantation. These effects are particularly significant in patients with multiple transfusions or repeated pregnancies. Consequently, identifying high-risk individuals, improving donor-recipient matching strategies, and developing early screening and intervention measures have become priorities in clinical practice.

Future research should focus on elucidating the mechanisms of RBC-induced immune tolerance, establishing integrative predictive models across disciplines, and developing precision transfusion strategies tailored to individual immune profiles. Such efforts will be essential for achieving effective control and intervention of RBC alloimmune responses.
